# Unzipping the defense: a comprehensive review on bZIP transcription factors in *Caenorhabditis elegans*

**DOI:** 10.3389/fcimb.2025.1673469

**Published:** 2025-10-01

**Authors:** Boopathi Balasubramaniam, Ashley V. Veatch, Ransome van der Hoeven

**Affiliations:** ^1^ Iowa Institute for Oral Health Research, University of Iowa College of Dentistry, Iowa City, IA, United States; ^2^ Department of Diagnostic and Biomedical Sciences, The University of Texas Health Science Center at Houston School of Dentistry, Houston, TX, United States; ^3^ Department of Periodontics, University of Iowa College of Dentistry, Iowa City, IA, United States

**Keywords:** *C. elegans*, bZIP transcription factors, innate immunity, nuclear localization, oxidative stress

## Abstract

*Caenorhabditis elegans* is a simple yet powerful host model organism for exploring how animals mount defenses against infection. In the absence of an adaptive immune system, it relies solely on innate immunity, making it an ideal model for studying pathogen-induced innate immune responses, which are often conserved across higher eukaryotic organisms. Among the numerous transcription factors encoded in the *C. elegans* genome, the basic leucine zipper (bZIP) family is particularly notable for its pivotal role in regulating immune and stress responses. Of the 29 major bZIP proteins identified in *C. elegans*, this review focuses on 12 that play a direct role in pathogen response and innate immunity. In this review, we summarize the basic structure and processing of bZIP proteins, explore their potential involvement in various pathways that regulate innate immune and stress responses, and highlight key scientific questions for future investigation. By shedding light on the complex yet coordinated immune strategies employed by *C. elegans* this review offers insights to enhance our understanding of innate immunity in more complex organisms, including humans.

## 1 Background

*C. elegans* has served as a key model organism in genetics and developmental biology for over 5 decades (Brenner, 1974; Nigon and Felix, 2017). Its utility spans both small- and large-scale experimental approaches, including RNA interference (RNAi) screening, CRISPR gene editing, and Green Fluorescent Protein (GFP) reporter assays (Fire et al., 1998; Corsi et al., 2015). The organism’s compact genome and proteome make it well suited for multi-omics approaches, enabling efficient exploration of complex biological networks with relatively modest resource requirements (Reece-Hoyes et al., 2013; Reinke et al., 2013). One of the impactful areas of *C. elegans* research is the study of host-pathogen interactions. In the natural environment, *C. elegans* is continuously exposed to a diverse array of microbes and has evolved mechanisms to discriminate between harmful pathogens and those that can serve as a nutritional source. Despite lacking adaptive immunity, *C. elegans* exhibit pathogen-specific responses and retains a form of immune memory from prior exposures. These characteristics establish *C. elegans* as a powerful model for elucidating fundamental host defense mechanisms and provide insight into the evolutionary origins of complex immune systems in higher organisms. The relevance stems from its conserved innate immune pathways, which share significant homology with those of mammals. Understanding these interactions is essential, as *C. elegans* provides unique advantages for dissecting innate immune mechanisms, including its genetic tractability, conserved signaling pathways, and well-established host-pathogen infection models. However, key molecular mechanisms remain unclear, especially those by which pathogens manipulate host cellular machinery to establish infection and promote disease progression (Balla and Troemel, 2013; Kumar et al., 2020).

As a valuable model organism, *C. elegans* possesses a simple yet robust immune system based solely on innate immunity. Its defense mechanisms engage highly conserved signaling pathways including the p38 mitogen-activated protein kinase (MAPK) cascade, insulin-like signaling, and the unfolded protein response (UPR) which are activated upon exposure to diverse pathogens (Balasubramaniam et al., 2019; Huang et al., 2020; Balasubramaniam et al., 2022; Duxbury et al., 2024). The innate immune pathways in *C. elegans* play a crucial role in regulating the synthesis of antimicrobial peptides and other immune effectors that combat invading pathogens during host-pathogen interactions. By activating these pathways, the worm effectively initiates and coordinates its defense mechanisms against various infections, underscoring the critical role of innate immunity in the host. Although *C. elegans* lack classical pattern recognition receptors, it detects pathogens through indirect sensing of infection-associated damage or physiological changes, triggering immune responses via conserved signaling pathways (Irazoqui et al., 2010). In some cases, specialized receptors such as NHR-86 and paired C-type lectins enable more direct recognition, triggering innate immune signaling via conserved mechanisms (Peterson et al., 2019; Liu et al., 2024). In addition, the nervous system plays a key role in pathogen detection and immune regulation in *C. elegans*. As previously noted, rather than relying on classical pattern recognition receptors and pathogen-associated molecular patterns, *C. elegans* detects microbial infections through disturbances in cellular homeostasis, a process known as surveillance immunity (Hahm et al., 2020; Wang et al., 2024). This strategy enables the nematode to recognize and respond to microbial exposure and invasion by sensing stress or damage signals. As the primary interface with pathogens, the gut, particularly the intestine plays a central role in the activation of these immune responses (Pukkila-Worley and Ausubel, 2012). In this context, epithelial tissues of *C. elegans* play a crucial role in host defense. The epidermis responds to extracellular pathogens, while the intestine targets gut-invading microbes. Despite lacking specialized immune cells such as dendritic cells and macrophages, these epithelial tissues can mount precise and effective immune responses, highlighting the organism’s reliance on non-cellular mechanisms for innate immunity (Pujol et al., 2008; Osman et al., 2018; Grover et al., 2021; Afridi and Tu, 2025). Collectively, the well-organized immune system in *C. elegans* establishes it as a powerful model for uncovering the evolutionary basis and conserved mechanisms of immunity across species (Martineau et al., 2021).

Among the many regulatory proteins involved in host defense responses, transcription factors play a central and coordinating role. By binding to specific DNA regions and modulating the transcriptional machinery, transcription factors enable precise regulation of gene expression in response to diverse stimuli (Spitz and Furlong, 2012). The *C. elegans* genome is estimated to encode approximately 900 transcription factors, as reported by various studies and databased such as WormBase (Reece-Hoyes et al., 2005; Craig et al., 2013; Narasimhan et al., 2015; Sternberg et al., 2024). This diverse group includes major families such as basic helix-loop-helix (bHLH), homeodomain, nuclear receptors, zinc finger proteins, and bZIPs (basic leucine zippers). Notably, the bHLH family has deep evolutionary roots, with homologs identified in early diverging eukaryotes such as algae and yeast, highlighting its ancient and conserved role in gene regulation. This suggests that these fundamental regulatory proteins emerged and diversified early in eukaryotic evolution, playing crucial roles in various biological processes across diverse lineages (Correa et al., 2008). There exists a significant gap in our understanding of innate immunity, particularly concerning the role of bZIP proteins, which remain underexplored in the context of host-pathogen interactions. Thus, this review examines the fundamental structure, activation mechanisms, post-translational processing, and the role of bZIP proteins in innate immunity.

## 2 bZIP transcription factors

Transcription factors are proteins that bind to specific DNA sequences near target genes to regulate their transcription into RNA which in turn play a crucial role in gene expression control (Afridi and Tu, 2025). They function as molecular switches, turning genes on or off in response to various signals, such as development, environmental changes, pathogen exposure, or cellular stress. By interacting with the promoter or enhancer regions of DNA upon various conditions, transcription factors recruit or block the transcriptional machinery, including RNA polymerase, thereby influencing gene expression patterns (Zhao and Emmons, 1995). Notably, dysregulation of transcription factors is often associated with diseases, including cancer and genetic disorders, making them important targets for biomedical research and therapeutic intervention in higher mammalian systems (Butt and Karathanasis, 1995; Vishnoi et al., 2020). Among the various transcription factors, bZIP proteins are a widespread family that play essential roles in regulating gene expression in response to a variety of cellular signals, including stress, immunity, metabolism, and development (Amoutzias et al., 2007; Afridi and Tu, 2025). A total of 29 bZIP proteins have been identified in the *C. elegans* genome, based on publicly available resources such as WormBase and WormBook (**Table 1**) (Sternberg et al., 2024). This review focuses on 12 bZIP transcription factors that play direct roles in host-pathogen interactions and innate immune defense in *C. elegans.*

**Table 1:** List of known bZIP transcription factors in *C. elegans*.

	Gene	UniProt ID	Description	Function	Subcellular localization	Phenotype on mutation	Human ortholog	References
1.	*atf-2*	Q21361	embryonic development, stress response	Enables RNA polymerase II transcription regulatory region sequence-specific DNA binding activity and protein heterodimerization activity. Involved in negative regulation of transcription by RNA polymerase II and positive regulation of transcription by RNA polymerase II.	Nuclear	fat content increased; gene expression level high	NFILZ/ NFIL3	(Erdelyi et al., 2011; Lemieux et al., 2011; Sternberg et al., 2024)
2.	*atf-4*	Q22156	amino acid metabolism, hydrogen sulfide biosynthetic process	Predicted to enable DNA-binding transcription activator activity, RNA polymerase II-specific and RNA polymerase II transcription regulatory region sequence-specific DNA binding activity. Involved in hydrogen sulfide biosynthetic process and positive regulation of gene expression.	Nuclear	–	ATF4/ ATF5	(Sternberg et al., 2024)
3.	*atf-6*	Q20435	ER stress sensor; UPR regulator; ER calcium ion homeostasis	Enables RNA polymerase II transcription regulatory region sequence-specific DNA binding activity. Involved in several processes, including ATF6-mediated unfolded protein response; determination of adult lifespan; and endoplasmic reticulum calcium ion homeostasis.	Nuclear, ER, membrane	early larval arrest; tunicamycin hypersensitive; shortened life span; pore forming toxin response variant	ATF6/ATF6B	(Shen et al., 2005; Bischof et al., 2008; Darom et al., 2010; Sternberg et al., 2024)
4.	*atf-7*	Q86MD3	innate immunity and stress response	Enables RNA polymerase II cis-regulatory region sequence-specific DNA binding activity and mitogen-activated protein kinase binding activity. Involved in several processes, including defense response to bacterium; regulation of innate immune response; and regulation of transcription by RNA polymerase II. Acts upstream of or within serotonin biosynthetic process.	Nuclear	pathogen induced gene expression variant; apoptosis reduced; transgene expression reduced	ATF2/ CREB5/ ATF7	(Richardson et al., 2010; Pukkila-Worley et al., 2012; Soltanmohammadi et al., 2022; Sternberg et al., 2024)
5.	*atf-8*	O16213	DNA binding; poorly characterized	Predicted to enable DNA-binding transcription factor activity, RNA polymerase II-specific and RNA polymerase II cis-regulatory region sequence-specific DNA binding activity. Predicted to be involved in regulation of transcription by RNA polymerase II.	Nuclear	–	TEF/DBP/HLF	(Sternberg et al., 2024)
6.	*atfs-1*	Q23272	Mitochondrial UPR regulator; stress response; innate immunity	Enables DNA-binding transcription factor activity, RNA polymerase II-specific; enzyme binding activity; and mitochondrial transcription factor activity. Involved in positive regulation of nematode larval development; regulation of gene expression; and response to stress.	Mitochondrial/ nuclear/ cytoplasm	chemical hypersensitive; developmental delay; protein phosphorylation increased; pathogen susceptibility increased; slow growth	–	(Baker et al., 2012; Nargund et al., 2012; Rauthan et al., 2013; Pellegrino et al., 2014; Sternberg et al., 2024)
7.	*cebp-1*	Q18909	innate immunity; regulation of extent of cell growth; regulation of presynapse assembly	Enables DNA-binding transcription factor activity, RNA polymerase II-specific; DNA-binding transcription factor binding activity; and RNA polymerase II cis-regulatory region sequence-specific DNA binding activity. Involved in several processes, including defense response to bacterium; regulation of extent of cell growth; and regulation of presynapse assembly.	Nuclear/ cytoplasm/ axon/ synapse	colchicine resistant; gene expression level reduced	–	(Bounoutas et al., 2011; Li et al., 2015; Sternberg et al., 2024)
8.	*cebp-2*	Q8IG69	innate immunity; lipid metabolism	Predicted to enable DNA-binding transcription factor activity, RNA polymerase II-specific and RNA polymerase II cis-regulatory region sequence-specific DNA binding activity. Involved in defense response to Gram-negative bacterium; regulation of lipid metabolic process; and regulation of transcription by RNA polymerase II.	Nuclear	Lethal; sterile; transgene expression reduced	CEBPB/ CEBPG	(MacNeil et al., 2015; Sternberg et al., 2024)
9.	*ces-2*	Q94126	apoptosis regulation	Enables RNA polymerase II transcription regulatory region sequence-specific DNA binding activity; identical protein binding activity; and protein heterodimerization activity. Involved in several processes, including apoptotic process; positive regulation of programmed cell death; and positive regulation of transcription by RNA polymerase II.	Nuclear	programmed cell death variant; spindle orientation variant; extended life span	TEF/HLF/DBP	(Hatzold and Conradt, 2008; Rozanov et al., 2020; Sternberg et al., 2024)
10.	*crh-1*	Q9U2I0	memory, development; dauer entry; determination of adult lifespan	Enables several functions, including DNA-binding transcription activator activity, RNA polymerase II-specific; identical protein binding activity; and transcription coactivator binding activity. Involved in several processes, including dauer entry; determination of adult lifespan; and positive regulation of transcription by RNA polymerase II.	Nuclear	associative memory defective; extended life span; cryophilic; transgene expression increased	–	(Nishida et al., 2011; Lakhina et al., 2015; Moss et al., 2016; Sternberg et al., 2024)
11.	*crh-2*	Q4JFH9	response to Gram negative bacterium (indirect); dauer regulation	Predicted to enable DNA-binding transcription factor activity, RNA polymerase II-specific and cAMP response element binding activity. Involved in defense response to Gram-negative bacterium; positive regulation of dauer entry; and regulation of gene expression.	Nuclear	exploded through vulva; linker cell migration variant	CREB3L2/ CREB3L1	(Lall et al., 2006; Schwarz et al., 2012; Sternberg et al., 2024)
12.	*fos-1*	G5ECG2	development, cell invasion; stress response	Enables several functions, including RNA polymerase II transcription regulatory region sequence-specific DNA binding activity; RNA polymerase II-specific DNA-binding transcription factor binding activity; and identical protein binding activity. Involved in several processes, including determination of adult lifespan; negative regulation of stress response to copper ion; and regulation of gene expression.	Nuclear	*Bacillus thuringiensis* toxin hypersensitive; anchor cell invasion variant; cell development variant	–	(Kao et al., 2011; Schindler and Sherwood, 2011; Ghosh and Sternberg, 2014; Sternberg et al., 2024)
13.	*jun-1*	G5ECU7	development, stress response	Enables RNA polymerase II-specific DNA-binding transcription factor binding activity and sequence-specific DNA binding activity. Involved in several processes, including determination of adult lifespan; positive regulation of RNA splicing; and response to starvation.	Nuclear	larval arrest; *Bacillus thuringiensis* toxin hypersensitive; embryonic lethal	JUNB/ JUN/ JUND	(Lehner et al., 2006; Ceron et al., 2007; Kao et al., 2011; Sternberg et al., 2024)
14.	*let-607*	O44743	protein folding, UPR, distal tip cell migration; innate immunity	Enables RNA polymerase II cis-regulatory region sequence-specific DNA binding activity. Involved in several processes, including protein folding; regulation of IRE1-mediated UPR; and regulation of distal tip cell migration.	Nuclear	dumpy; apoptosis regulation; RAB-11 recycling endosome morphology variant	CREB3/CREB3L3/ CREB3L4	(Fraser et al., 2000; Chen et al., 2008; Winter et al., 2012; Venz et al., 2020; Sternberg et al., 2024)
15.	*skn-1*	P34707	oxidative stress response; innate immunity; macromolecule biosynthetic process	Enables Hsp70 protein binding activity and RNA polymerase II cis-regulatory region sequence-specific DNA binding activity. Involved in several processes, including endoderm development; positive regulation of macromolecule biosynthetic process; and response to stress.	Nuclear/ mitochondrion/ cytoplasm/ ER	ER stress response variant; chemical hypersensitive; embryonic development variant; embryonic lethal; larval lethal; fungal resistance increased, etc.,	NFE2L2/ NFE2L1/ NFE2/ NFE2L3	(Maduro et al., 2007; Mosbech et al., 2013; Lehrbach and Ruvkun, 2016; Grover et al., 2024; Sternberg et al., 2024)
16.	*sknr-1*	A0A0M7REQ4	predicted to be involved in regulation of transcription by RNA polymerase II	Predicted to enable DNA-binding transcription factor activity, RNA polymerase II-specific and RNA polymerase II cis-regulatory region sequence-specific DNA binding activity. Predicted to be involved in regulation of transcription by RNA polymerase II.	Nuclear	embryonic lethal; dauer lifespan extended; mRNA surveillance defective	NFE2L2/ NFE2L1/ NFE2/NFE2L3	(Simmer et al., 2003; Sun Y. et al., 2011; Xie and Roy, 2012; Sternberg et al., 2024)
17.	*xbp-1*	G5EE07	ER stress; UPR regulator; determination of adult lifespan	Enables RNA polymerase II transcription regulatory region sequence-specific DNA binding activity. Involved in several processes, including determination of adult lifespan; positive regulation of transcription by RNA polymerase II; and response to stress. Acts upstream of with a positive effect on negative regulation of multicellular organismal process.	Nuclear	ER stress response variant; axon regeneration defective; developmental delay; apoptosis increased	XBP1	(Shen et al., 2001; Urano et al., 2002; Arsenovic et al., 2012; Liu et al., 2020; Sternberg et al., 2024)
18.	*zip-1*	Q9Y0B9	innate immunity	Predicted to enable DNA binding activity.	–	transgene expression reduced	–	(Gang et al., 2022; Lazetic et al., 2022; Sternberg et al., 2024)
19.	*zip-2*	Q21148	innate immunity; macromolecule biosynthetic process	Predicted to enable DNA-binding transcription factor activity, RNA polymerase II-specific and RNA polymerase II cis-regulatory region sequence-specific DNA binding activity. Involved in defense response to bacterium and positive regulation of macromolecule biosynthetic process.	Nuclear	chemical resistant: pathogen induced gene expression variant; pathogen susceptibility increased	–	(Estes et al., 2010; Pellegrino et al., 2014; Sternberg et al., 2024)
20.	*zip-3*	Q9XUK2	innate immunity; UPR	Predicted to enable DNA-binding transcription activator activity, RNA polymerase II-specific and RNA polymerase II transcription regulatory region sequence-specific DNA binding activity. Involved in UPT^mt^ and negative regulation of innate immune response.	Nuclear	–	–	(Deng et al., 2019; Sternberg et al., 2024)
21.	*zip-4*	H2L0N3	innate immunity	Predicted to enable DNA-binding transcription factor activity, RNA polymerase II-specific and RNA polymerase II cis-regulatory region sequence-specific DNA binding activity. Involved in defense response to Gram-negative bacterium.	Nuclear	–	–	(Tjahjono and Kirienko, 2017; Sternberg et al., 2024)
22.	*zip-5*	G5EBE5	regulation of transcription by RNA polymerase II	Predicted to enable DNA-binding transcription factor activity, RNA polymerase II-specific and RNA polymerase II cis-regulatory region sequence-specific DNA binding activity. Predicted to be involved in regulation of transcription by RNA polymerase II.	–	associative olfactory memory phenotype: plate tap reflex variant	–	(Swierczek et al., 2011; Cohen and Rabinowitch, 2024; Sternberg et al., 2024)
23.	*zip-6*	G5EFX5	regulation of transcription by RNA polymerase II	Predicted to enable DNA-binding transcription factor activity, RNA polymerase II-specific and RNA polymerase II cis-regulatory region sequence-specific DNA binding activity. Predicted to be involved in regulation of transcription by RNA polymerase II.	Nuclear	lethal; sterile; transgene expression reduced	–	(MacNeil et al., 2015; Sternberg et al., 2024)
24.	*zip-7*	Q9NAE4	DNA-templated transcription and regulation of DNA-templated transcription	Enables identical protein binding activity. Predicted to be involved in DNA-templated transcription and regulation of DNA-templated transcription.	–	transgene expression reduced	–	(MacNeil et al., 2015; Sternberg et al., 2024)
25.	*zip-8*	P46505	regulation of transcription by RNA polymerase II	Predicted to enable DNA-binding transcription factor activity, RNA polymerase II-specific and RNA polymerase II cis-regulatory region sequence-specific DNA binding activity. Predicted to be involved in regulation of transcription by RNA polymerase II.	–	lethal; sterile	–	(Sternberg et al., 2024)
26.	*zip-9*	A0A1I6CMA8	regulation of transcription by RNA polymerase II	Predicted to enable DNA-binding transcription activator activity, RNA polymerase II-specific and RNA polymerase II transcription regulatory region sequence-specific DNA binding activity. Predicted to be involved in regulation of transcription by RNA polymerase II.	Nuclear	–	–	(Sternberg et al., 2024)
27.	*zip-10*	Q22835	regulation of gene expression; innate immunity	Enables core promoter sequence-specific DNA binding activity. Involved in regulation of gene expression.	Nuclear	long, rays fused	BATF3	(Liang et al., 2007; Irfan Afridi et al., 2023; Sternberg et al., 2024)
28.	*zip-11*	Q9N5A8	innate immunity	Enables DNA-binding transcription factor binding activity. Involved in defense response to Gram-negative bacterium.	Nuclear	–	–	(Sternberg et al., 2024)
29.	*zip-12*	Q9XW80	DNA binding; poorly characterized	Enriched in several structures, including anterior hypodermis; intestine; mechanosensory neurons; rectum; and uterus based on microarray; tiling array; RNA-seq; and single-cell RNA-seq studies. Is affected by several genes including *cyc-1*; *nuo-6*; and *etr-1* based on microarray and RNA-seq studies. Is affected by thirteen chemicals including 1-methylnicotinamide; levamisole; and Zidovudine based on RNA-seq and microarray studies.	–	tail morphology variant	–	(Jafari et al., 2011; Sternberg et al., 2024)

## 3 Structure and organization of bZIPs

**Figure 1:**
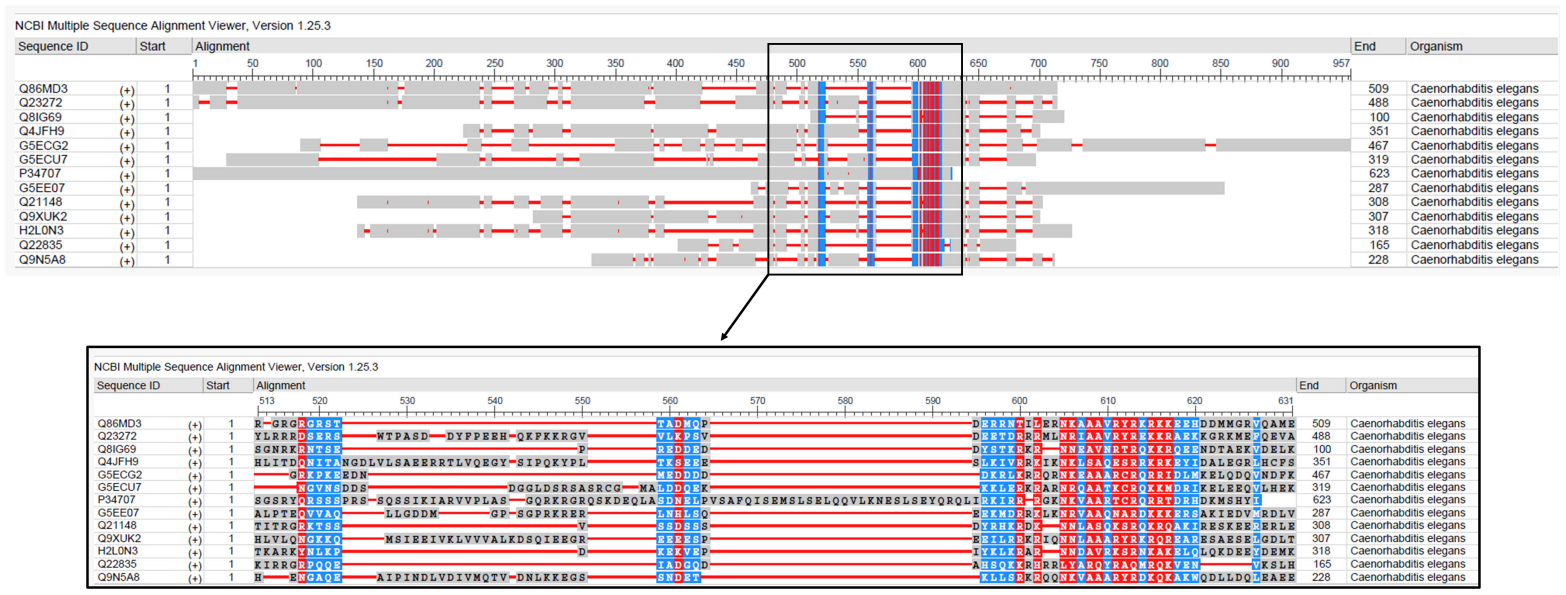
Multiple sequence alignment of bZIP proteins involved in pathogen response and innate immunity in *C. elegans*. The alignment displays conserved residues. Sequences were aligned using NCBI MSA viewer.

**Figure 2:**
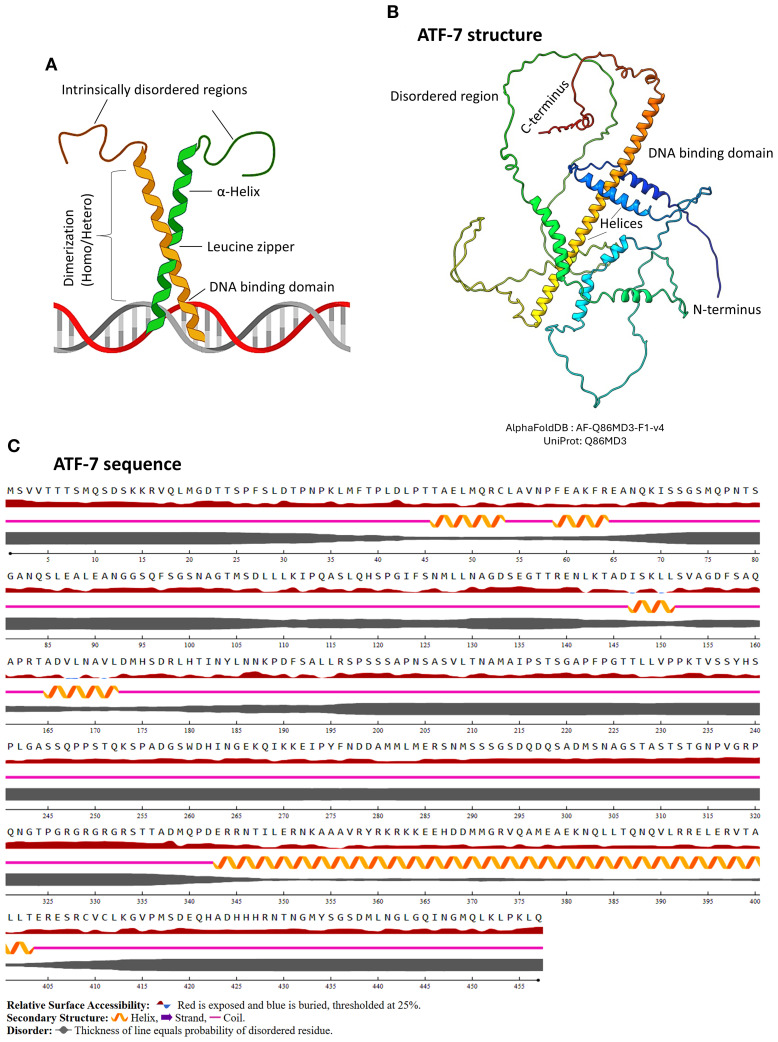
Basic structural features of bZIP protein. **(A)** Schematic representation of a bZIP protein, highlighting the α-helical regions, DNA-binding domain, and the leucine zipper motif. The figure was generated using BioRender software. **(B)** Predicted 3D structure of the representative bZIP protein ATF-7, generated using AlphaFold and visualized in ChimeraX. ATF-7 is one of the major well studied bZIPs in *C*. *elegans* due to its functional relevance under certain conditions including host-pathogen interactions and innate immunity. The structure illustrates the characteristic bZIP fold, including the DNA-binding and dimerization regions. **(C)** Annotated amino acid sequence of ATF-7, showing domain organization. The image was rendered using NetSurfP-3.0, indicating helices, zipper region, intrinsically disordered regions, and secondary structure elements.

Protein alignment of the selective proteins reveals limited similarity outside the conserved bZIP domain, suggesting that these factors are subject to distinct regulatory mechanisms. The presence of disordered regions and structural variability further indicates functional flexibility in their organization (**Figure 1**). The defining feature of these proteins is their bZIP domain, which consists of two key parts: a basic region that binds DNA, and a leucine zipper that enables dimerization (**Figure 2**). The basic region contains positively charged amino acids like lysine and arginine, which facilitate interaction with negatively charged DNA backbone. The leucine zipper, on the other hand, is made up of repeating leucine or other hydrophobic residues at every seventh position. These repeating pattern forms an amphipathic α-helix that allows two similar or different bZIP proteins to come together in a coiled structure either as homodimer or heterodimer. Dimerization is essential in bZIP organization because only the paired form can effectively bind DNA and regulate gene expression (Vinson et al., 1993). The ability to form diverse dimer combinations greatly expands their functional repertoire, enabling precise and flexible control over gene expression in response to environmental and physiological cues (Fujii et al., 2000; Miller, 2009). This flexible structural organization explains why bZIP transcription factors play such diverse and tightly regulated roles across eukaryotes.

## 4 Subcellular localization of bZIPs

The subcellular localization of bZIP proteins is tightly regulated, as their function depends on being targeted to the correct destination under specific physiological conditions. Although their primary site of action is the nucleus, where they bind specific DNA sequences to regulate gene expression, these proteins are initially synthesized and localized in the cytoplasm. In many cases, the conformationally unstable bZIP monomers are retained in the cytoplasm in an inactive form, either due to lack of dimerization, or masking of their nuclear localization signals (NLS) (Inoue et al., 2005). Upon activation by stimuli such as pathogens, stress, or metabolic signals, bZIP proteins dimerize through their leucine zipper domains either as homo- or heterodimer. A classic example in *C. elegans* is the ZIP-2 protein, which remains in the cytoplasm under normal conditions but translocates to the nucleus during bacterial infection, where it activates genes involved in the immune response (Kahn et al., 2008; Estes et al., 2010). This regulatory mechanism ensures that bZIPs respond precisely to environmental and developmental cues by altering their subcellular localization in a signal-dependent manner.

## 5 Activation and processing of bZIPs

**Figure 3:**
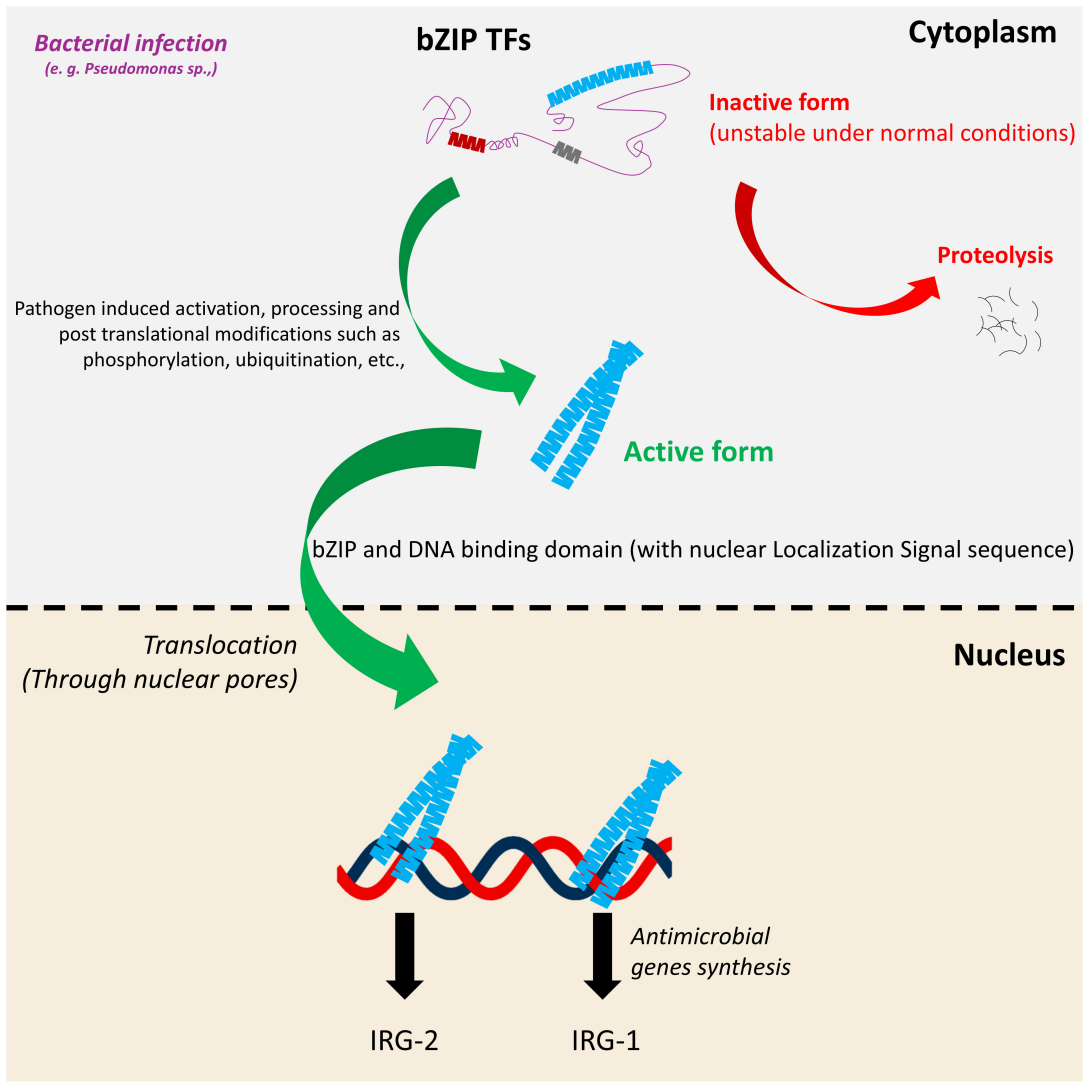
Generalized processing and activation mechanism of bZIP proteins in response to pathogen exposure. Under normal conditions, bZIP proteins remain in an inactive or unstable form in the cytoplasm. Upon pathogen exposure, they undergo specific PTMs such as cleavage and/or phosphorylation which stabilize the protein and enable its activation. The processed bZIP protein translocates to the nucleus, where it binds to target DNA sequences and activates the transcription of antimicrobial genes or stress responsive genes. This regulatory cascade contributes to the host’s defense response against pathogen invasion including *Pseudomonas* sp.

Under normal conditions, bZIPs remain inactive in the cytoplasm, primarily due to absence of dimerization. However, upon bacterial infection in *C. elegans*, intracellular signaling cascades rapidly promote their stabilization, dimerization, and accumulation. For example, during *Pseudomonas aeruginosa* infection, global translation is inhibited, yet ZIP-2 paradoxically accumulates post-translationally in the cytoplasm, enabling an immune response despite the translational blockade (Dunbar et al., 2012). There, ZIP-2 undergoes dimerization and translocates to the nucleus through nuclear pores to initiate the antimicrobial gene expression (**Figure 3**). The canonical nuclear import pathway involves importins (heterodimers/homodimers) recognizing NLSs in these proteins. This complex is then translocated through nuclear pore complexes via the Ran-GTP cycle, facilitating regulated entry into the nucleus (Malinow et al., 2019). A noteworthy representative example is CEBP−1, which is critical for axon regeneration in *C. elegans*. It is identified to have three NLS motifs within its sequence that collectively facilitate nuclear import. Mechanistically, one of these signal motifs directly binds to Importin−α (IMA−3), mediating its transport through nuclear pores (Malinow et al., 2019)., Apart from CEBP-1, the nuclear localization mechanisms of other bZIP transcription factors in *C. elegans* remain largely unexplored. Hence, CEBP-1 stands out as the well-characterized model for nuclear import among bZIPs in *C. elegans*. Upstream triggers such as pathogen exposure or cellular stress can induce the nuclear localization of bZIP transcription factors, enabling them to activate downstream target genes via their NLS. However, the duration of their nuclear residence remains poorly understood and may be tightly regulated to ensure precise temporal control of gene expression. Overall, bZIP transcription factors are regulated through mechanisms that govern their subcellular localization and post-translational modifications (PTMs), with NLSs playing a significant role in their activation. These signals ensure that bZIP proteins translocate to the nucleus only under specific conditions, such as during exposure to pathogens or cellular stress, thereby enabling context-dependent regulation of gene expression.

## 6 Post translational modifications of bZIPs

Several bZIP proteins in *C. elegans*, including ZIP-2, ATF-7, and SKN-1, are regulated through PTMs that fine-tune their activity in response to environmental stress. Phosphorylation is one of the predominant PTMs affecting the activation of bZIP factors; for example ATF-7 is phosphorylated by the p38-MAPK (PMK-1), leading to a functional switch from a repressor to an activator of immune genes during pathogen infection (**Table 2**) (Shivers et al., 2010). Functional studies using a phosphorylation-defective mutant *atf-7* (qd22) demonstrated that loss of phosphorylation disrupts this switch, severely impairing the expression of PMK-1 target genes and reducing pathogen resistance. Although the work provides compelling evidence of ATF-7 phosphorylation through mobility shifts observed in immunoblots, it does not identify the specific amino acid residues or phosphorylation sites targeted by PMK-1 (Shivers et al., 2010). Likewise, SKN-1 is regulated by phosphorylation via kinases such as GSK-3 and PMK-1, affecting its nuclear localization and transcriptional activity under oxidative or xenobiotic stress (Hilgarth et al., 2003; An et al., 2005; Hwang et al., 2022). While modifications such as ubiquitination, or acetylation have been shown to modulate the activity, stability, or DNA-binding function of bZIP transcription factors in other organisms, their roles in regulating bZIP proteins including ATF-7, SKN-1, and others remain largely unexplored (Hilgarth et al., 2003; Izumiya et al., 2005; Campbell and Izumiya, 2012). Interestingly, SUMOylation has been studied in several *C. elegans* bZIP transcription factors, including SKN-1 and XBP-1 (Lim et al., 2014; Princz et al., 2020). For instance, one study demonstrated that the SUMO-specific peptidase ULP-4 modulates the mitochondrial UPR (UPR^mt^) by regulating the transcription factor ATFS-1. During mitochondrial stress, ATFS-1 undergoes SUMOylation at lysine 326 (K326), and ULP-4-mediated deSUMOylation enhances its stability and transcriptional activity. This regulation is essential for activating the UPR^mt^, promoting innate immunity, and extending lifespan (Gao et al., 2019). Despite these findings, other key PTMs of *C. elegans* bZIP proteins remain unexplored. While bZIP proteins primarily function as DNA-binding transcription factors, their activity may also be influenced by the chromatin remodeling, including histone modifications such as methylation and acetylation. These epigenetic changes can alter DNA accessibility, potentially modulating bZIP binding and function (Cusack et al., 2020). However, the extent to which such PTMs of histones affect bZIP-mediated transcriptional regulation in *C. elegans* remains uncharacterized. Therefore, understanding the spectrum and functional consequences of PTMs in *C. elegans* bZIP proteins is crucial for deciphering their dynamic roles in immune signaling and cellular homeostasis.

**Table 2:** Innate immunity-associated bZIP transcription factors and their regulatory pathways.

	Gene name	Pathogen	Pathways involved	Expression	Curated PTMs	References
1	*atf-7*	*Pseudomonas* sp.,	p38-MAPK	body wall musculature; hypodermis; intestine; neurons; rectal epithelial cell	Phosphorylation	(Shivers et al., 2010; Pukkila-Worley et al., 2012; Kishore et al., 2024; Sternberg et al., 2024)
2	*atfs-1*	*Pseudomonas* sp.,	UPR^mt^	Ubiquitous	SUMOylation	(Pellegrino et al., 2014; Gao et al., 2019; Sternberg et al., 2024; Xiao et al., 2024a)
3	*cebp-1*	*Pseudomonas* sp.,	cAMP Pathway; JNK and p38-MAPK pathways; TGF-β/Smad signaling pathway	intestine; muscle cell; neurons; pharynx; somatic cell	–	(Li et al., 2015; McEwan et al., 2016; Tjahjono and Kirienko, 2017; Malinow et al., 2019; Robert et al., 2020; Sternberg et al., 2024)
4	*cebp-2*	*Pseudomonas* sp.,	p38 MAPK pathway; lipid metabolism	somatic cell	–	(Xu et al., 2015; Tjahjono and Kirienko, 2017; Sternberg et al., 2024)
5	*skn-1*	*Pseudomonas* sp.,; *Enterococcus faecalis;* microsporidia; oomycete	p38-MAPK pathway; insulin/IGF-like signaling pathway; proteasome surveillance pathway; intracellular pathogen response (IPR) pathway	germ line; head ganglion; intestine; neurons; pharynx	Phosphorylation (S74, S164, S340, S393, S397, S430, S432); SUMOylation	(Inoue et al., 2005; Tullet et al., 2008; Hoeven et al., 2011; Princz et al., 2020; Wu et al., 2021; Grover et al., 2024; Sternberg et al., 2024)
6	*xbp-1*	*Pseudomonas* sp.,; *Bacillus thuringiensis*	UPR^mt^; p38-MAPK pathway	Ubiquitous	SUMOylation	(Bischof et al., 2008; Richardson et al., 2010; Richardson et al., 2011; Lim et al., 2014; Dai et al., 2015; Sternberg et al., 2024)
7	*zip-1*	Orsay virus; *Nematocida parisii; Myzocytiopsis humicola*	IPR pathway	–	–	(Gang et al., 2022; Lazetic et al., 2022)
8	*zip-2*	*Pseudomonas* sp.,	Translational surveillance and immune activation pathway	Pharynx; intestine	–	(Estes et al., 2010; Dunbar et al., 2012; Tjahjono and Kirienko, 2017; Vasquez-Rifo et al., 2020; Sternberg et al., 2024)
9	*zip-3*	*Pseudomonas* sp.,	UPR^mt^	pharyngeal muscle; head mesodermal cell; intestine; sensory neuron; muscle cell	–	(Deng et al., 2019; Sternberg et al., 2024)
10	*zip-4*	*Pseudomonas* sp.,	UPR^mt^	gonad; pharynx; spermatheca; vulva	–	(Tjahjono and Kirienko, 2017; Sternberg et al., 2024)
11	*zip-10*	*Pseudomonas* sp.,	insulin/IGF-1 signaling pathway; p38-MAPK pathway	pharyngeal muscle cell; neurons	–	(Lee et al., 2021; Irfan Afridi et al., 2023; Sternberg et al., 2024)
12	*zip-11*	*Pseudomonas* sp.,	p38-MAPK pathway	Intestine; pharynx; hypodermis	–	(Zheng et al., 2021; Sternberg et al., 2024)

## 7 Dynamics of bZIPs

The dynamics of bZIP transcription factors in *C. elegans* reflect a specific regulatory network that responds rapidly to diverse stimuli, including pathogen infection, oxidative stress, and xenobiotic exposure. While the roles of many *C. elegans* bZIP proteins in various biological processes are well-established, the dynamic and context-specific nature of their activation, particularly the rapid kinetics of their nuclear translocation and PTMs remains less thoroughly investigated (Zhuang et al., 2022). However, these dynamics comprehend changes in localization, stability, DNA-binding activity, and interactions with co-factors, allowing bZIP proteins to function as both activators and repressors depending on the context. The timing and scale of these responses are often transient and reversible, highlighting the importance of PTMs and feedback regulation in modulating bZIP activity (Sun J. et al., 2011). Importantly, crosstalk with other signaling pathways such as insulin-like signaling, TGF-β, and UPR pathways along with the innate immune pathway further add layers of regulation that ensure context-specific outcomes.

## 8 Turnover of bZIPs

bZIP transcription factors and their turnover mechanism in *C elegans* are a significantly regulated process that ensures precise control of their activity in response to physiological and environmental signals to maintain homeostasis (**Figure 3**). Protein turnover, primarily mediated by the ubiquitin-proteasome system, plays a crucial role in regulating the abundance and activity of bZIP factors such as SKN-1. For example, SKN-1 is subjected to proteasomal degradation under normal conditions, a process mediated by E3 ubiquitin ligases that tag the protein for proteolysis, thereby preventing inappropriate activation of stress-response genes. Upon oxidative stress or pathogenic challenge, stabilization of SKN-1 occurs through inhibition of its proteasomal degradation, often mediated by upstream kinase signaling, such as PMK-1, which disrupts interactions with ubiquitination machinery (Lehrbach and Ruvkun, 2016; Turner et al., 2024). Although direct evidence for proteasomal turnover of other *C. elegans* bZIP proteins is limited, transcriptional and translational control mechanisms suggest that regulated protein degradation is likely to contribute to the transient nature of their immune responses. Additionally, the interplay between turnover and PTMs such as phosphorylation and ubiquitination may dictate both the stability and functional significance of these bZIP proteins. Despite their importance, detailed mechanisms of bZIP turnover in *C. elegans* remain poorly studied, representing a key area for future research into the spatiotemporal regulation of innate immunity.

## 9 bZIPs in *C. elegans* immunity and pathogen defense

bZIP transcription factors play a critical role in sensing and transducing signals related to stress and infection, thereby regulating the expression of immune effector genes, including those encoding antimicrobial proteins. Through diverse mechanisms including phosphorylation, nuclear translocation, and interaction with other signaling pathways such as the p38-MAPK cascade, bZIPs facilitate highly effective and specific defense strategies against different pathogens. The following section highlights several bZIP transcription factors and their roles in pathogen exposure and innate immunity.

### 9.1 ATF-7

ATF-7 is a cAMP-dependent transcription factor that acts downstream of the p38-MAPK signaling pathway in *C. elegans*, a key regulator of innate immunity in response to pathogen exposure and stress response. ATF-7 is an ortholog of human ATF2, ATF7, and CREB5. ATF-7 controls the transcription of immune defense related genes, enabling the worm to produce an effective response against microbial infection (Fletcher et al., 2019). Notably, studies have shown that ATF-7 also plays a vital role in long-term stress responses, suggesting that the innate immune response in *C. elegans* is integrated with the cellular stress response (Shivers et al., 2010). This dual role implies that immune activation triggered by pathogens is likely part of a broader stress response system. Within this immune-stress response cascade, PMK-1 and ATF-7 work together to coordinate defenses against biotic and environmental abiotic threats (Ren and Ambros, 2015; Hall et al., 2017; Wu et al., 2019). Moreover, a study demonstrated that upon pathogen infection, such as with *P. aeruginosa*, the kinase PMK-1 phosphorylates the bZIP transcription factor ATF-7 converting it from a transcriptional repressor into an activator. This modification enables the upregulation of a broad array of immune-related genes, thereby enhancing the worm’s defense mechanisms (Shivers et al., 2010). These findings reveal a conserved and interconnected signaling network where innate immunity and stress adaptation converge. This highlights the pivotal role of ATF-7 as a central mediator in preserving *C. elegans* homeostasis under adverse environmental conditions, such as pathogen exposure.

### 9.2 ATFS-1

ATFS-1 is an activating transcription factor associated with stress responses and is the *C. elegans* ortholog of the human ATF4 gene (Melber and Haynes, 2018). It plays a key role in immune defense through its regulation of the mitochondrial unfolded protein response (UPR^mt^). Recent research has shown that activating the UPR^mt^ either genetically or with compounds like caffeic acid can enhance immune function in *C. elegans* (Xiao et al., 2024a). Also, several studies suggest a role for activating mitochondrial pathways via mild stress and immune protection (Pellegrino et al., 2014; Deng et al., 2019; Yang et al., 2022). Upon mitochondrial stress, ATFS-1 accumulates in both the mitochondria and the nucleus, where it regulates the expression of genes involved in mitochondrial maintenance and function. This dual localization is essential for the effective activation of UPR^mt^ and the restoration of mitochondrial homeostasis (Nargund et al., 2015). When *C. elegans* encounters bacterial pathogens such as *Pseudomonas* sp., mitochondrial stress can be induced, leading to activation of ATFS-1 (Deng et al., 2019). Upon activation, ATFS-1 translocates to the nucleus and induces the expression of genes that enhance both mitochondrial homeostasis and immune defense. This coupling of mitochondrial stress responses with innate immunity suggests that ATFS-1 plays a key role in coordinating the organism’s ability to respond effectively to the infection by enhancing resilience against pathogen-induced damage.

### 9.3 CEBP-1

CEBP-1 is a homologous to the mammalian C/EBP (CCAAT/enhancer-binding protein) family, which is broadly involved in immune regulation and cellular stress responses (Kim et al., 2002). In *C. elegans*, CEBP-1 plays a crucial yet nuanced role in modulating the innate immune response to pathogenic bacteria, including *P. aeruginosa* (McEwan et al., 2016; Tjahjono and Kirienko, 2017). While many immune-regulatory bZIP transcription factors in *C. elegans* such as ZIP-2 and CEBP-2 function as positive regulators, CEBP-1 functions more as a context-dependent negative regulator of innate immunity. CEBP-1’s role is particularly prominent in the intestinal immune surveillance pathway, where it modulates host responses to bacterial toxins and infection. A recent study demonstrated that loss of *cebp-1* activity can enhance survival in *C. elegans* exposed to *P. aeruginosa* or its virulence factor, Exotoxin A (ToxA). In mutants lacking *nipi-3*, a kinase-related protein that normally represses *cebp-1*, elevated *cebp-1* activity resulting in immune suppression and increased susceptibility to infection. However, mutating *cebp-1* in this background restores immune function and pathogen resistance, indicating that overactivation of CEBP-1 can reduce protective immunity (McEwan et al., 2016). Thus, CEBP-1 acts a negative regulator of innate immunity in *C. elegans*. Although, *cebp-1* is not directly required for the induction of classic pathogen-response genes such as *irg-1*, *irg-2*, *clec-60*, its regulatory influence modulates the threshold and timing of immune activation during bacterial infection. CEBP-1 interacts with several key proteins to regulate diverse biological processes. For example, it forms a complex with ETS-4 following axon injury (Li et al., 2015). Additionally, CEBP-1 binds to the importin-α protein IMA-3, which mediates its nuclear import, a process critical for axon regeneration in adults, although not required for initial axon development (Malinow et al., 2019). These interactions highlight CEBP-1’s multifaceted roles in transcriptional regulation, stress response, and neuronal repair in *C. elegans* extending beyond its involvement in innate immunity.

### 9.4 CEBP-2

The gene *cebp-2* encodes a *C. elegans* ortholog of human CCAAT/enhancer-binding protein gamma (C/EBPγ), with well-established roles in lipid metabolism and immune surveillance (Xu et al., 2015; Reddy et al., 2016). Particularly, CEBP-2 regulates fatty acid β-oxidation, a mitochondrial process essential for energy production, and *cebp-2*-deficient worms exhibit reduced lifespan compared to wild-type animals (Tjahjono and Kirienko, 2017; Hahm et al., 2020). In the context of innate immunity, CEBP-2 plays a key role during pathogen exposure. It functions downstream of, or in parallel with, key signaling pathways such as the p38-MAPK pathway, modulating the expression of antimicrobial peptides and other immune effector genes. Notably, the immune surveillance pathway involving CEBP-2 is also linked to mitochondrial function, suggesting a mechanistic overlap between metabolic regulation and immunity (Tjahjono and Kirienko, 2017). Studies on *C. elegans* innate immunity have shown that CEBP-2 functions primarily as a heterodimer, often partnering with ZIP-2 and other bZIP family transcription factors. This dimerization is crucial for modulating the expression of antimicrobial peptides and defense-related genes during pathogen infection, allowing appropriate regulation of immune responses (Estes et al., 2010; Reddy et al., 2016; Tjahjono and Kirienko, 2017; Hahm et al., 2020; Zheng et al., 2021). Evidence from RNAi knockdowns and mutant analyses indicates that CEBP-2 can act both redundantly with other bZIP transcription factors and via distinct transcriptional targets, ensuring a context-specific immune response (Reddy et al., 2016). Overall, CEBP-2 serves as a key integrator of metabolic state and pathogen defense, coordinating mitochondrial function with transcriptional control of immunity.

### 9.5 SKN-1

The *skn-1* gene encodes the *C. elegans* homolog of mammalian NFE2 and Nrf2, a well-studied transcription factor with over 600 publications in PubMed, notably in lifespan regulation, endoplasmic reticulum (ER) UPR (UPR^ER^), and metabolism. In addition, SKN-1 plays a crucial role in innate immunity, improving survival after infection (Wu et al., 2021). Its expression declines with age, offering insights into the weakening of immune responses in aging organisms (Kim et al., 2022). Activated by oxidative or xenobiotic stress, SKN-1 induces genes involved in antioxidant defense, phase II detoxification, and proteostasis (Blackwell et al., 2015). Beyond stress response, SKN-1 enhances innate immunity by upregulating protective genes during pathogen exposure, which in turn reduces the oxidative damage caused by infection and inflammation. This response supports both cellular health and longevity under stress (Inoue et al., 2005; Papp et al., 2012; Goh et al., 2023). SKN-1 activation is controlled by phosphorylation events mediated by upstream kinases such as the p38-MAPK and GSK-3, which modulate its nuclear translocation and transcriptional activity, and it is a well-established mechanism. Upon activation, SKN-1 induces a network of downstream effectors including phase II detoxification enzymes such as glutathione S-transferases (GSTs) and NADPH quinone oxidoreductase (NQO-1), which mitigate oxidative stress and restore cellular redox balance. SKN-1 also directly activates innate immune response genes, such as those encoding antimicrobial peptides and C-type lectins, which help neutralize pathogens and limit infection-induced damage (Blackwell et al., 2015). Additionally, SKN-1 regulates proteostasis by upregulating components of the ubiquitin-proteasome system and molecular chaperones, thereby enhancing the cell’s capacity to manage damaged proteins during stress (Li et al., 2011; Choe and Leung, 2013). SKN-1 is expressed in multiple isoforms, each exerting distinct regulatory roles in stress responses and immunity. For instance, both SKN-1A and SKN-1C isoforms are critical for oxidative stress resistance, highlighting their protective function in contexts where detoxification of reactive oxygen species is essential. Although SKN-1C mutants have not been directly tested in infection assays, available evidence indicates that SKN-1A and SKN-1C may function redundantly to support bacterial immunity, suggesting overlapping roles in host defense (Papp et al., 2012; Hu et al., 2017; Jochim et al., 2025). In contrast, loss of SKN-1A, which primarily localizes to the ER and mediates responses to proteasome dysfunction, enhances resistance to natural pathogens such as oomycetes and microsporidia (Grover et al., 2024). A study demonstrated that SKN-1A, the ER-localized isoform of SKN-1, requires the AAA^+^ ATPase CDC-48 and ER proteostasis machinery for its activation during bacterial infection, highlighting an isoform-specific mechanism of immune regulation in *C. elegans* (Gabaldon et al., 2024). These contrasting phenotypes establish that SKN-1 isoforms can differentially modulate immune responses depending on the pathogen encountered and the physiological state of the host (Gabaldon et al., 2024; Nair et al., 2024; Jochim et al., 2025). Collectively, these findings underscore that SKN-1 does not function as a uniform regulator, but rather as an isoform-specific hub whose immune outcomes are shaped by the dynamic interplay among isoform expression, pathogen type, and the cellular stress environment.

### 9.6 XBP-1

The *xbp-1* gene in *C. elegans*, an ortholog of human XBP1, encodes a key transcription factor involved in immune defense, lifespan regulation, and the UPR^ER^ (Richardson et al., 2010; Tillman et al., 2018). Under ER stress, the sensor IRE-1 splices *xbp-1* mRNA, producing an active form of XBP-1 that translocates to the nucleus and activates genes responsible for protein folding, quality control, and degradation to maintain homeostasis. This phenomenon is critical not only for maintaining ER homeostasis but also for enhancing innate immunity during infection. Beyond its role in ER stress, XBP-1 has been shown to be important for innate immunity as well in *C. elegans*, particularly during pathogen exposure. Activation of the UPR via XBP-1 enhances the organism’s ability to survive infection by supporting the secretory pathway and promoting the expression of immune-related genes (Richardson et al., 2010; Richardson et al., 2011). Specifically, during *P. aeruginosa* PA14 infection, the microRNA mir-233 is upregulated in a p38-MAPK-dependent manner and suppresses the ER Ca²^+^-ATPase SCA-1, thereby activating the IRE-1/XBP-1 branch of the UPR, which is essential for innate immunity (Dai et al., 2015). Similarly, exposure to the pore-forming toxin Cry5B from *Bacillus thuringiensis* induces ER stress and requires XBP-1–mediated UPR for host defense, highlighting a conserved, toxin-responsive role for XBP-1 in protecting *C. elegans* against bacterial challenge (Bischof et al., 2008; Richardson et al., 2010). Additionally, compounds such as Dioscin have been shown to enhance innate immunity and promote pathogen avoidance in *C. elegans* by activating the XBP-1 pathway, suggesting the potential for therapeutic modulation of immune responses (Xiao et al., 2024b). This links cellular stress responses with immune defense mechanisms, illustrating how *C. elegans* integrate environmental and pathogen-induced stresses to maintain homeostasis through the XBP-1 cascade.

### 9.7 ZIP-1

ZIP-1 is one of the bZIPs that plays a pivotal role in the innate immune response of *C. elegans*, particularly through its regulation of the intracellular pathogen response (IPR). Recent research has identified ZIP-1 as a key regulator necessary for the induction of *pals-5* and other genes involved in the IPR pathway. Upon activation of the IPR, typically triggered by pathogen infection (Orsay virus and *Nematocida parisii*) or cellular stress, ZIP-1 protein levels increase. Functionally, ZIP-1 acts as a transcriptional activator, directly promoting the expression of *pals-5*, a gene integral to the organism’s innate immunity mechanism against intracellular pathogens. The significance of ZIP-1 in this process is underscored by studies using *zip-1* mutants, where the induction of *pals-5*p::GFP IPR is significantly reduced. This phenotype demonstrates that the absence of *zip-1* significantly compromises the IPR in *C. elegans* (Lazetic et al., 2022). Another study demonstrated that a gain-of-function mutation in *pals-25* triggers two immune programs: the Oomycete Recognition Response (ORR) in the epidermis and the IPR in the intestine. These responses provide protection against the oomycete *Myzocytiopsis humicola*, the microsporidian parasite *N. parisii*, and viral infections such as the Orsay virus. The study shows that ZIP-1 is crucial for activating subsets of IPR genes, particularly in the epidermis where it localizes to the nucleus upon activation. Interestingly, immune activation in the epidermis can communicate with the intestine, establishing a systemic immune response that enhances resistance to multiple pathogens (Gang et al., 2022). To date, ZIP-1 research has focused on intracellular pathogens like *N. parisii* and the Orsay virus, which activate the IPR. In contrast, the role of ZIP-1 in the *C. elegans* response to bacterial infection remains unclear and needs further investigation.

### 9.8 ZIP-2

In *C. elegans*, the *zip-2* gene encodes a transcription factor with a bZIP domain, likely related to the human ATF2 family, though it lacks a direct human ortholog. Among bZIP family members, ZIP-2 is one of the extensively studied, particularly for its role in innate immunity and its interaction with CEBP-2. Unlike the classical p38-MAPK pathway, ZIP-2 mediates an alternative immune signaling pathway specifically activated during *P. aeruginosa* infection (Vasquez-Rifo et al., 2020). ZIP-2 translocates to the nucleus in response to infection and induces immune effector genes, including *irg-1*, which help the organism fight off pathogens (Estes et al., 2010; Dunbar et al., 2012). Mechanistically, *P. aeruginosa* releases Exotoxin A, which disrupts host protein synthesis by inhibiting mRNA translation in the intestinal epithelium. Interestingly, rather than suppressing the immune response, this stress activates specific immune effectors, including the transcription factor ZIP-2. Under normal conditions, ZIP-2 is translationally repressed due to the presence of an upstream open reading frame (uORF) in its 5′ untranslated region (UTR), which destabilizes the mRNA and blocks efficient translation. However, when global translation is suppressed by the toxin, this repression is alleviated, permitting the synthesis of ZIP-2. Once synthesized, ZIP-2 becomes activated, enters the nucleus through nuclear pores, and initiates the transcription of immune response genes such as *irg-1*. This mechanism allows *C. elegans* to maintain immune defense even under conditions of translational stress (Estes et al., 2010). Beyond its role in innate immunity, ZIP−2 also functions as a broader cellular surveillance factor, responding to translational inhibition and mitochondrial stress independent of pathogen infection. Activation of ZIP−2 under these conditions triggers transcriptional programs that maintain cellular homeostasis, delay aging-associated phenotypes, and promote organismal resilience, highlighting its role as a general stress-responsive regulator in *C. elegans* (Hahm et al., 2020).

### 9.9 ZIP-3

The gene *zip-3* encodes an ortholog of human ATF5. Although ZIP-3 is less extensively studied than ZIP-2, evidence suggests that ZIP-3 participates in modulating innate immune responses, mainly under conditions of pathogen exposure and cellular stress. ZIP-3 is believed to repress UPR^mt^ response by negatively regulating ATFS-1. The authors suggest that *P. aeruginosa* exploit this interaction to impair mitochondrial function and immune response (Deng et al., 2019; Anderson and Haynes, 2020). ZIP-3 may function either independently or in coordination with other immune pathways to regulate the expression of defense genes, contributing to the worm’s ability to respond to microbial infection and maintain homeostasis (McEwan et al., 2016; Deng et al., 2019). Although ZIP-3 has not been extensively studied in the context of immunity, it may contribute to the host defense response, potentially working alongside other bZIP transcription factors such as ATFS-1 which is known to mediate responses to mitochondrial stress and infection.

### 9.10 ZIP-4

While ZIP-4 is less characterized among the bZIPs, a study suggested that it contributes to the transcriptional control of genes involved in defense against *Pseudomonas* sp (Tjahjono and Kirienko, 2017)., and external stimuli. ZIP-4 likely functions within the complex network of transcription factors that coordinate immune gene expression in response to microbial infection, helping *C. elegans* possess an effective defense. Its activity reflects the broader role of bZIP proteins in mediating immune and stress-related transcriptional regulation rather than a specific role. ZIP-4 contributes to the regulation of stress responses and immune defense by sensing mitochondrial stress and is believed to function in coordination with ZIP-2, CEBP-2, and CEBP-1 (Tjahjono and Kirienko, 2017). However, ZIP−4’s specific interactions with other bZIPs such as heterodimerization or direct binding have not been mechanistically elucidated yet.

### 9.11 ZIP-10

ZIP-10 is another important bZIP transcription factor that has been studied in the context of various stress conditions and immune responses in *C. elegans*. ZIP-10 has no well-characterized direct ortholog in humans, but it is part of a conserved family of bZIP transcription factors, which includes multiple human orthologs with similar domains such as the basic leucine zipper ATF-like transcription factor 3 (BATF3). ZIP-10 functions as a negative regulator of innate immunity, in part by repressing the p38-MAPK pathway. This attenuation of PMK-1 activity leads to reduced expression of immune effectors, highlighting ZIP-10’s role as a key modulator that balances immune responses to prevent overactivation (Irfan Afridi et al., 2023). In a study, researchers discovered that communication between Gamma-Aminobutyric Acid (GABA) signaling between the gut associated neurons and smooth muscle triggers the release of a neuropeptide FLP-6. FLP-6, in turn suppresses the activity of two transcription factors, ZIP-10 and KLF-1, within the intestinal lining. This enhances the innate immune response through the depression of the PMK-1/p38 pathway. Overall, this study reveals a neuroimmune axis where GABAergic signaling, FLP-6, ZIP-10, and KLF-1 coordinate to protect the gut from *Pseudomonas* infection (Liu et al., 2023). Furthermore, ZIP-10 interacts with the transcription factor SKN-1, implicating it in immune aging and reinforcing the link between immune and stress response pathways. Specifically, ZIP-10 modulates SKN-1-dependent targets by forming a positive feedback loop with INS-7 and the insulin/IGF-1 signaling (IIS) pathway, promoting immunosenescence in aged *C. elegans*. Interestingly, suppressing ZIP-10 leads to reduced INS-7, enhanced SKN-1 activity, and improved pathogen resistance in older worms (Hahm et al., 2020; Lee et al., 2021; Kim et al., 2022). Although ZIP-10 is part of a broader network of bZIP transcription factors that coordinate the expression of antimicrobial peptides and other immune effectors, its specific upstream regulators, downstream targets, and molecular partners remain largely unknown. Understanding these interactions will be essential to fully elucidate ZIP-10’s role in coordinating stress resistance and immune regulation.

### 9.12 ZIP-11

The gene *zip-11* encodes a member of the bZIP transcription factor family that exhibits limited similarity to the human JUN protein. Researchers discovered that ZIP-11 is upregulated in the intestine upon infection and is essential for host resistance. For example, ZIP-11 operates through two distinct mechanisms, one of which involves establishing a feedback loop with the conserved p38 MAPK signaling pathway. This interaction enhances the innate immune response by amplifying signaling and transcriptional activation during infection. The second mechanism involves interaction with CEBP-2: ZIP-11 forms a complex with the CCAAT/enhancer-binding protein CEBP-2 to mediate transcriptional responses to infection independently of the p38-MAPK pathway. This alternative pathway highlights the versatility of ZIP-11 in regulating immune gene expression through distinct signaling routes. Additionally, the study demonstrates that the human homolog ATF4 can be a functional substituted for ZIP-11 in regulating innate immunity in *C. elegans*. These findings suggest that the ZIP-11/ATF4 genetic program activates native innate immune responses through conserved PMK-1/p38 and CEBP-2 immune signals in *C. elegans*, potentially reflecting a similar regulatory mechanism in other organisms (Zheng et al., 2021). Its functional conservation with human ATF4 suggests that studying ZIP-11 can provide valuable insights into evolutionarily conserved mechanisms of immune regulation.

## 10 Human orthologs of *C. elegans* bZIP transcription factors

Many transcription factors in *C. elegans* do not appear to have identifiable orthologs. However, it is noted that a few human orthologs of *C. elegans* bZIP factors have been implicated in innate immunity (Liang et al., 2011; Ebert et al., 2022). For example, human ortholog of ATF-7, which is ATF7, regulates stress responses, including those mediated by the UPR and inflammatory signaling pathways. ATF7 modulates innate immune responses by controlling the expression of pro-inflammatory cytokines and stress-responsive genes (Yoshida et al., 2015). Likewise, Human ortholog of SKN-1: Nrf2 (NFE2L2) is a critical regulator of the antioxidant response and plays a vital role in innate immunity by controlling the expression of genes involved in redox homeostasis, detoxification, and immune response. Nrf2 activation enhances resistance to pathogens and inflammation by upregulating antioxidant enzymes and immune-related genes through p38-MAPK pathway (Hammad et al., 2023; Xiao et al., 2025). Likewise, human ATF4 can substitute for ZIP-11 in *C. elegans*, highlighting the evolutionary conservation of immune regulatory pathways (Zheng et al., 2021). Further studies on these bZIP transcription factors in the worm could provide important insights into how the p38-MAPK pathway, the UPR, and other signaling pathways work together to combat pathogens. Moreover, if orthologous genes exist in humans, this line of research could help identify novel therapeutic targets for immune-related disorders and infectious diseases.

## 11 Experimental approaches for studying transcription factors

To investigate the function, DNA-binding activity, and regulatory roles of transcription factors, a variety of molecular and genetic techniques are available. For example, Chromatin immunoprecipitation (ChIP) and ChIP-Seq are widely used to find DNA binding sites across the genome, providing insights into their target genes and regulatory networks. While electrophoretic mobility shift assays (EMSA) help to detect direct DNA-transcription factor interactions. Reporter genes such as GFP, red fluorescent protein, luciferase, etc., are useful to study the localization and expression patterns (Fried and Crothers, 1981; de Wet et al., 1987; Johnson et al., 2007). Complementing these approaches, Targeted DNA adenine methyltransferase Identification (Targeted DamID or TaDa) enables tissue-specific profiling of transcription factor binding *in vivo* without the need for cell isolation. TaDa has been successfully used in *C. elegans* to identify direct targets of transcription factors such as LIN-22 and NHR-25 in the epidermis, revealing their roles in antagonizing stem cell fate and providing a detailed view of gene regulatory networks in a physiologically relevant context. As such, TaDa represents a promising and emerging approach for transcription factor profiling, with significant potential for systematically mapping bZIP family proteins in *C. elegans* (Greil et al., 2006; van den Ameele et al., 2019; Katsanos and Barkoulas, 2022). Similarly, Spec-seq is a high-throughput technique that quantitatively profiles DNA-binding preferences of transcription factors, enabling precise identification of their target sequences. While extensively demonstrated in mammals, its application in *C. elegans* remains limited (Narasimhan et al., 2015; Stormo et al., 2015; Zuo et al., 2022). Additionally, protein expression and localization can also be examined using Western blotting and immunofluorescence microscopy, respectively. Functional studies often involve gene knockdown or knockout approaches such as RNA interference (RNAi) or CRISPR-Cas9 genome editing to assess the impact on phenotypes. Protein-protein interactions involving transcription factors can be investigated using yeast two-hybrid systems, co-immunoprecipitation or proximity labelling (Hazafa et al., 2020; Zhang et al., 2021; Schutz et al., 2022; Binti et al., 2024). Proteomics offers a powerful approach to investigate global changes in protein expression resulting from mutations in individual or multiple transcription factors. Further supporting this, integrative omics technologies such as transcriptomics, metabolomics, and epigenomics can provide deeper insights into the regulatory networks governed by these factors. Furthermore, advanced biophysical techniques like DNA foot printing and surface plasmon resonance enable precise characterization of transcription factor-DNA interactions, revealing exact binding sites and affinities (Galas and Schmitz, 1978; Fujii et al., 2000). Bioinformatics/motif analysis can be used to predict target genes and binding motifs using known bZIP consensus sequences (Reinke et al., 2013). Together, these complementary or integrative techniques allow a comprehensive understanding of transcription factors in *C. elegans* and in various organisms under different contexts including pathogen exposure.

## 12 Translational relevance of *C. elegans* bZIP transcription factors

From a translational perspective, *C. elegans* provides an excellent platform to represent how bZIP factors mediate host responses to toxins, pathogens, and oxidative stress conditions relevant to human diseases including infection, neurodegeneration, inflammation, and cancer. For instance, the well-studied bZIP protein SKN-1, the ortholog of mammalian Nrf2, is essential for oxidative stress resistance and detoxification, with clear parallels to Nrf2-driven cytoprotective responses in humans. Similarly, ATF-7 is regulated by p38-MAPK, mimics stress-induced transcription factor regulation in mammalian innate immunity (An et al., 2005; Shivers et al., 2010). Because of the genetic tractability and short lifecycle of *C. elegans*, bZIP-related pathways can be rapidly manipulated *in vivo* through CRISPR/Cas9 editing, RNAi, or reporter assays to test conserved gene regulatory circuits under various conditions including host-pathogen interactions. High-throughput drug screening in *C. elegans* using bZIP-responsive reporters has also revealed potential modulators of inflammation and oxidative damage, facilitating early-phase identification of therapeutic candidates (Tullet et al., 2008). Besides, the conservation of bZIP domains and downstream target genes enables cross-species comparative genomics, which in turn enhances the value of *C. elegans* based studies for mammalian systems. Thus, translational research in *C. elegans* bZIPs holds significant promise for identifying conserved regulatory networks and therapeutic targets relevant to human disease contexts.

## 13 Discussion

Since *C. elegans* relies exclusively on innate immunity to detect and respond to microbial pathogens, this defense is primarily mediated by conserved signaling pathways and a complex network of transcription factors. Key transcription factors such as ATF-7, SKN-1, DAF-16, and HLH-30 act downstream of well-studied pathways including p38-MAPK and insulin/IGF-1 signaling to regulate antimicrobial and stress response genes (Ogg et al., 1997; Inoue et al., 2005; Shivers et al., 2010; Hoeven et al., 2011; Visvikis et al., 2014; Naji et al., 2018; Balasubramaniam et al., 2022). Notably, due to its compact genome, *C. elegans* often repurpose the same signaling pathways to carry out multiple biological functions. Evidences suggest that the UPR^ER^ and UPR^mt^ serve as key stress signals, activating host defense mechanisms in response to a variety of environmental and pathogenic stressors (Richardson et al., 2010; Pellegrino et al., 2014; Tillman et al., 2018; Soo et al., 2021). In response to pathogen exposure, *C. elegans* exhibit avoidance behavior mediated by neuronal pathways involved in sensory processing. This suggests that environmental sensing in *C. elegans* involves crosstalk between neuronal circuits and immune defense pathways (Meisel and Kim, 2014; Cao et al., 2017; Singh and Aballay, 2020). bZIPs have been implicated in diverse biological processes beyond gene regulation, including development and neuronal function. For example, *cebp-2* mutants exhibit reduced body size and altered fat metabolism, suggesting a potential role in metabolic and growth regulatory pathways (Reddy et al., 2016). Interestingly, in *C. elegans*, bZIPs can function as positive (e.g. ATF-7, and ZIP-2) and negative regulators (CEBP-1, and ZIP-3) of innate immunity (Shivers et al., 2010; Reddy et al., 2016; Lee et al., 2021; Irfan Afridi et al., 2023). However, the molecular mediators that link innate immune pathways with stress-related pathways and transcription factors remain incompletely understood. Further research is needed to elucidate their precise mechanisms of action and signaling cascades that mediate the crosstalk between innate immune and stress response pathways. Collectively, the complexity and versatility of the innate immune system in *C. elegans*, where transcription factors such as bZIPs integrate multiple signaling pathways to combat infection underscore the importance of studying these regulatory networks in a greater detail.

## 14 Gaps in knowledge and future directions

**Figure 4:**
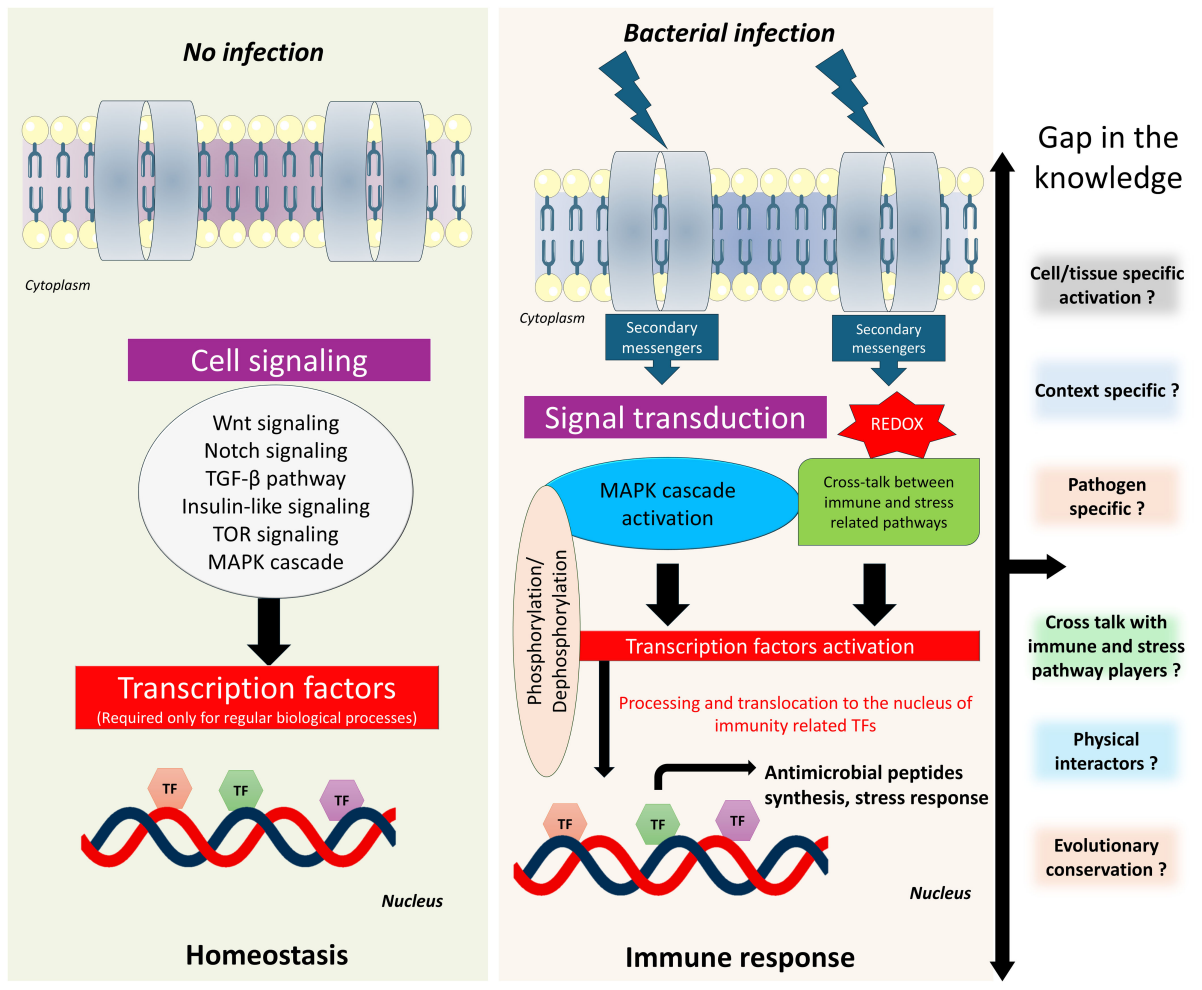
Schematic representation of host immune response regulation in *C. elegans*. The diagram shows how bZIP transcription factors coordinate immune responses to infection in *C. elegans* upon infection compared to normal conditions. bZIP activation is influenced by multiple signaling cascades, including the p38-MAPK pathway and potentially other parallel or intersecting pathways. Crosstalk between these pathways is context-dependent and remains unclear. The other key questions remain unresolved such as the involvement of specific upstream regulators, how different pathogens modulate bZIP activity, and the nature of context-specific protein-protein interactions.

Despite growing insights into the functions of bZIP transcription factors in *C. elegans* immunity, various important questions are still unresolved (**Figure 4**). A key open question is whether bZIP proteins contribute to immune memory-like phenomena in *C. elegans*, such as trained immunity, a form of non-genetic, inducible immune adaptation. It remains unclear whether pathogens produce effector molecules that can directly inhibit or hijack bZIP activity as a strategy for immune evasion, or whether intermediary factors mediate this interaction. While bZIP factors may act redundantly, it is unknown whether simultaneous knockdown of multiple bZIPs leads to synthetic lethality or functional compensation upon pathogen exposure and normal physiological conditions. From an evolutionary perspective, the extent to which immune-regulatory bZIP functions are conserved among *C. elegans*, *Drosophila*, and mammals is still under investigation; however, it is possible that bZIP pathways have diverged or adapted specifically in *C. elegans* due to their unique ecological niches. It also remains unclear whether mammalian bZIP orthologs can functionally substitute their *C. elegans* counterparts *in vivo* which may open a new avenue in immunology and microbiology. Mechanistically, the interplay between key immune signaling pathways including p38-MAPK, DAF-2/Insulin, and the UPR calls for deeper investigation. Persistent questions remain regarding the ability of bZIPs to form heterodimers with other regulators such as CEBP-2 or ATF-7, and whether their activity is modulated or repressed by additional transcriptional regulators during infection. Moreover, the role of bZIP proteins in interacting with chromatin remodelers and epigenetic modifiers such as histones during immune responses and normal physiological conditions remains poorly understood. Currently, the physical interactors of most innate immune related bZIP transcription factors in the worm remain unidentified, as no studies have yet reported such interactions. Additionally, the temporal dynamics of bZIP activation, processing, and nuclear translocation whether occurring during early, transient, or late phases of the immune response are still not clearly defined and may vary depending on the nature of the pathogenic exposure. These processes are context-specific, further complicating our understanding. Therefore, many unanswered questions remain regarding the roles and regulation of bZIP transcription factors in *C. elegans*, highlighting the need for further investigation into their functional networks and regulatory mechanisms. In addition to established methodologies, emerging technologies such as CRISPR, Spec-seq, single-cell omics, and ChIP-seq offer powerful tools for dissecting the function of bZIP transcription factors in *C. elegans*. For instance, Spec-seq has been used to demonstrate that BATF3, a mammalian ortholog of *C. elegans* ZIP-10 forms heterodimers with JUNB, recognizes specific DNA motifs, and exhibits conserved DNA-binding specificity, suggesting evolutionary conservation in bZIP-mediated immune regulation (Chang et al., 2018; Wang et al., 2025). Studying bZIP transcription factors using these advanced technologies offers the potential to systematically map target genes, uncover protein–protein interactions, and elucidate regulatory mechanisms involved in pathogen responses in *C. elegans*. Additionally, comparative analyses with orthologous proteins across species may reveal conserved functional roles. Together, these approaches promise to illuminate bZIP regulatory networks, uncover functional redundancy, and highlight evolutionarily conserved mechanisms of immune regulation.

## 15 Conclusion

bZIP transcription factors are an essential and versatile family of regulatory proteins that coordinate the innate immune and stress responses in *C. elegans*. Their conserved structural features such as leucine zipper regions and DNA binding domains, dynamic localization, and complex regulatory mechanisms enable precise control of gene expression during pathogen exposure and other stimuli. Despite significant technological advancements, many questions remain unanswered regarding the specific target genes of bZIP transcription factors, their interaction networks, and their crosstalk with other immune regulators during pathogen exposure. Future research using emerging omics approaches, gene editing, and imaging technologies will be crucial to unravel the full spectrum of bZIP functions and their evolutionary significance in innate immunity. Understanding these host defense mechanisms in response to external stimuli not only deepens our knowledge of *C. elegans* biology but may also provide insights into conserved immune strategies relevant to higher organisms.
